# The Evolving Role of Cryosurgery in Breast Cancer Management: A Comprehensive Review

**DOI:** 10.3390/cancers15174272

**Published:** 2023-08-26

**Authors:** Kefah Mokbel, Alevtina Kodresko, Heba Ghazal, Ramia Mokbel, Jon Trembley, Hussam Jouhara

**Affiliations:** 1 The London Breast Institute, Princess Grace Hospital, London W1U 5NY, UK; 2 Heat Pipe and Thermal Management Research Group, College of Engineering, Design and Physical Sciences, Brunel University, London UB8 3PH, UK; 3 School of Pharmacy and Chemistry, Kingston University, Kingston Upon Thames KT1 2EE, UK; 4 The Princess Grace Hospital, Part of HCA Healthcare UK, London W1U 5NY, UK; 5 Air Products PLC, Hersham Place Technology Park, Molesey Road, Surrey KT12 4RZ, UK; 6 Vytautas Magnus University, Studentu Street 11, LT-53362 Akademija, Kaunas District, Lithuania

**Keywords:** breast cancer, cryogenic media, cryosurgery, cryoablation, nitrogen

## Abstract

**Simple Summary:**

Breast cancer is the most prevalent cancer and the second leading cause of death among women, primarily due to the development of deadly metastases in 25–50% of patients, resulting in an unfavorable prognosis. Consequently, there is growing interest in exploring innovative therapeutic approaches to improve clinical outcomes and enhance the quality of life for breast cancer patients. One such approach that shows promise is the application of ultra-low cryogenic temperatures through cryosurgery. This review aims to critically examine the current understanding and state-of-the-art practices of cryosurgery as a treatment for breast cancer, while also addressing the challenges and prospects associated with this approach. By conducting a comprehensive literature review, this paper intends to provide an updated and comprehensive perspective on the topic.

**Abstract:**

Breast cancer is the most commonly diagnosed type of cancer, accounting for approximately one in eight cancer diagnoses worldwide. In 2020, there were approximately 2.3 million new cases of breast cancer globally, resulting in around 685,000 deaths. Consequently, there is an ongoing need to develop innovative therapeutic approaches that can improve both clinical outcomes and patient quality of life. The use of ultra-low cryogenic temperatures, facilitated by cryogenic media such as liquid nitrogen, has revolutionized the biomedical field and opened up new possibilities for advanced clinical treatments, including cryosurgery. Cryosurgery has demonstrated its feasibility as a minimally invasive technique for destroying breast tumors and eliciting a significant antitumor immune response in the host. This feature sets cryosurgery apart from other ablative techniques. It has been shown to be well tolerated and effective, offering several advantages such as simplicity, the avoidance of general anesthesia, minimal pain, low morbidity, short recovery time, cost-effectiveness, and notably, improved aesthetic outcomes. The reviewed studies indicate that cryosurgery holds promise in the management of early-stage breast cancer and metastatic disease, especially in triple-negative and Her2-positive molecular subtypes in conjunction with checkpoint inhibitors and anti-Her2 antibodies, respectively. Furthermore, the effectiveness of cryosurgery in the management of ductal carcinoma in situ should be investigated as an alternative modality to surgery or surveillance. The minimally invasive nature of cryosurgery has the potential to significantly enhance the quality of life for patients.

## 1. Introduction

Cryogenic Media and Biomedical Applications

Since its inception in 1877, cryogenics has made remarkable progress in both theory and experimentation, leading to significant advancements in the production and behavior of materials at extremely low temperatures. These advances have paved the way for various applications of cryogenic heat transfer, revolutionizing modern technology. Cryogenic media, also referred to as cryogens, now play a critical role in aerospace, healthcare, advanced manufacturing, machining methods, superconductivity, and advanced scientific research [1]. Notably, cryogenic media, such as nitrogen (N_2_), helium-3 (3He), argon (Ar), and oxygen (O_2_), have found extensive use in the biomedical sector due to their distinctive properties and material characteristics. These cryogens have boiling points of −195.79 °C, −269.96 °C, −185.85 °C, and −182.96 °C, respectively. N_2_ and He are particularly favored due to their inert behavior, favorable thermal properties, widespread availability, and the cost effectiveness of liquid nitrogen. Furthermore, helium-3’s ability to exist in its liquid phase at temperatures close to absolute zero makes it valuable, albeit challenging to process and store efficiently. Argon serves as an inert component in cryogenic separation units during the fractional distillation of liquid air, while oxygen, despite its reactivity, instability, and strong oxidizing features, is commercially viable and readily accessible as a cryogen [2,3]. Ongoing research efforts are focused on developing new numerical models to investigate the mechanisms of heat transfer and material behavior related to cryogens. These studies are crucial for enhancing the performance of cryogenic technologies and for exploring innovative and efficient methods of energy storage in processing and storage systems.

Over the past few years, there has been a significant increase in experimental and clinical research focusing on cryogenic techniques that utilize liquid nitrogen (N_2_) for the treatment of various medical conditions. These techniques have shown promise in the management of urologic tumors such as prostate and renal cancer, gastroenterological conditions like Barrett’s esophagus, and breast malignancies. The results obtained from these studies are encouraging, as they demonstrate low rates of recurrence, progression, and complications, while also minimizing morbidity and ensuring acceptable safety profiles. Moreover, these cryogenic methods are robust, minimally invasive, and user-friendly, offering great potential for improving cancer management and enhancing patients’ quality of life.

Considering the high prevalence of breast cancer, this article aims to provide a comprehensive and current review of the potential role of cryotherapy in breast cancer management. It will explore existing knowledge gaps and propose necessary limitations to be addressed in future research. By doing so, this article seeks to contribute to the advancement of cryotherapy as a valuable therapeutic option for breast cancer treatment.

## 2. Cryobiology

The use of low temperatures in healthcare has a rich history dating back to 2500 BC in ancient Egypt, where the concept of using cold to treat injuries and inflammation originated [4]. Centuries of research and remarkable discoveries have led to the development of cryosurgery, a cutting-edge medical technique. Cryosurgery involves the use of a cryoprobe, a small vacuum-insulated tube-like tool that serves as a conduit for a cryogenic liquid, causing complete tissue destruction. It is important to note that cryosurgery, or cryoablation, should not be confused with cryotherapy, which refers to non-invasive treatments involving the application of N_2_ vapor or air refrigeration at temperatures around −160 °C. Cryotherapy can be applied locally or as whole-body cryotherapy (WBC), which involves briefly exposing the entire body to temperatures ranging from −110 °C to −160 °C in a chamber environment.

**Figure 1:**
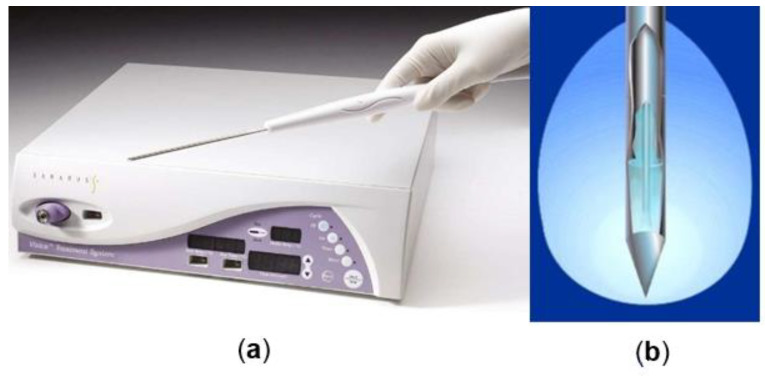
(**a**) Percutaneous cryoablation probes. (**b**) Schematic representation of the active tip of a cryoablation probe and the ice ball formed [5,6].

Significant advancements in cryogenic technologies have given rise to third-generation cryosurgery systems, which operate based on the principle of temperature change accompanying gas expansion without heat exchange with the environment. These modern systems utilize intra-operative imaging in real-time to monitor the precise placement of ultra-small cryoprobes and the extent of the freezing process. This enables easier and less traumatic probe placement, greater control over treatment margins, and ensures a more uniform temperature distribution, resulting in more efficient heat transfer. By rapidly expanding argon (Ar) or nitrogen (N_2_) gas through the cryoprobe’s tip, these systems can achieve extreme temperature drops, freezing adjacent tissues to approximately −140 °C within seconds. This leads to the formation of an ice ball and causes cell death within approximately 3 mm beyond the margins of the ball, as illustrated in Figure 1 [5].

**Figure 2:**
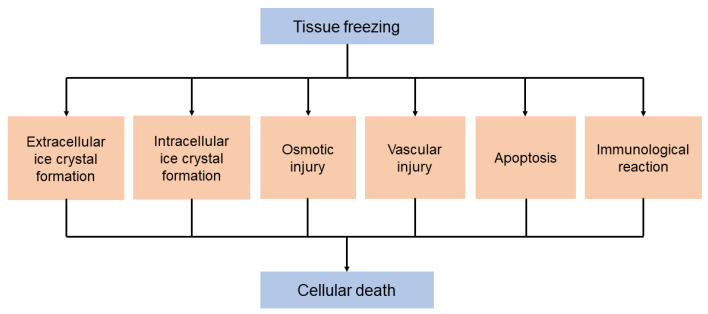
Underlying pathophysiological mechanisms of the destructive effects of cryosurgery on biological tissues and the associated tissue damage.

The subsequent thawing is achieved through the instillation of helium gas. Cryosurgery has demonstrated its effectiveness as a less invasive alternative with lower morbidity rates compared with other surgical resection techniques for treating serious health conditions like cancer. Figure 2 provides a schematic representation of the underlying pathophysiological mechanisms involved in the destructive effects of cryosurgery on biological tissues and the resulting tissue damage.

**Figure 3:**
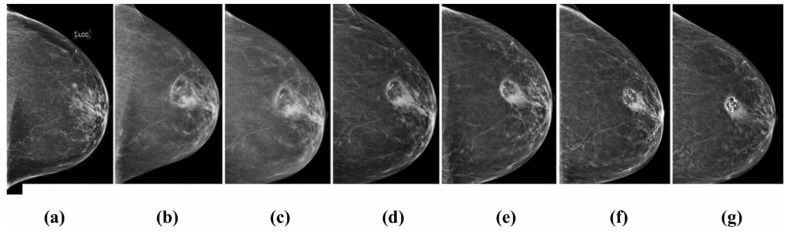
Mammography imaging of a left breast with a 0.5 cm invasive ductal carcinoma with tubular features demonstrating the resolving zone of fat necrosis around the area treated by cryoablation: (**a**) Before cryoablation; and (**b**) 6 months; (**c**) 12 months; (**d**) 24 months; (**e**) 36 months; (**f**) 48 months; (**g**) 60 months after cryoablation [9].

Experimental studies have provided evidence that direct cellular injury occurs due to the formation of extracellular ice crystals, leading to the development of a concentration gradient. This gradient causes enzymatic damage and denaturation of proteins. Additionally, in the case of rapid freezing, intracellular crystal aggregation disrupts mitochondria and organelles, ultimately resulting in cell membrane rupture during the thawing process [7]. As a delayed reaction, the release of toxic free radicals from hyperemic injury reperfusion has been recorded, which induces peroxidation of the cell-wall lipid membrane. Vascular injury, as another delayed mechanism, has been observed to occur hours or days later, when intracellular ice crystals damage the endothelium. This damage leads to vascular stasis, platelet aggregation, and the formation of intravascular thrombus, resulting in ischemia and tissue necrosis. Over time, this necrotic tissue is gradually reabsorbed [8]. Please refer to Figure 3 for a visual representation of these processes.

Furthermore, it has been suggested that cells not directly destroyed by cryofreezing undergo apoptosis, typically at the periphery of the cryoprobe ice ball. Moreover, ultra-low temperatures can induce immune-mediated cytotoxicity, involving the infiltration and activities of macrophages, neutrophils, and T cells, leading to subsequent tumor cell death. However, it is important to note that the effectiveness of cryosurgery can vary and be less controllable, as the degree of cell injury varies for each cell in a tissue, and cells react differently to various thermal histories, including cooling temperature and rate, duration of exposure, final temperature, and hold time.

While extensive studies have been conducted, demonstrating the current understanding of cryobiology mechanisms through in vitro and in vivo experiments, importantly, there are still knowledge gaps, specifically in the immune response in humans, that warrant further research. The complex nature of the cryosurgery process and the existence of multiple theories hinder further advancements in this field. Therefore, additional research is needed to address these gaps and to facilitate future developments.

### 2.1. Cryosurgery and Breast Cancer

Breast cancer is currently the most common malignancy and the second leading cause of death among women, mainly due to the development of deadly metastases in 25–50% of patients, resulting in an unfavorable prognosis. Only 25% of patients have a 5-year survival rate [10]. Traditional surgical methods for removing the primary tumor, such as breast-conserving surgery or total mastectomy, often require general anesthesia and hospitalization, and are associated with postoperative pain and potential adverse cosmetic effects.

To address these issues, minimally invasive modalities have gained significant interest. These modalities allow for the ablation of benign (e.g., fibroadenoma) or malignant breast lesions under local anesthesia in an outpatient setting. They cause minimal pain, promote quicker recovery, and result in better cosmetic outcomes with minimal tissue loss, smaller wounds, and less breast deformity. The availability of commercial liquefied gases has revolutionized modern cryosurgery, offering novel and state-of-the-art treatment options for patients with breast cancer [11,12].

Furthermore, advancements in screening and imaging technologies have led to the detection of breast cancers at earlier stages, resulting in a better prognosis with 5-year survival rates ranging from 98% to 99%. However, current treatment protocols have been associated with overtreatment and unnecessary surgical interventions, emphasizing the need to de-escalate breast cancer surgery toward less invasive approaches. The American College of Surgeons has outlined three essential criteria for any novel treatment modality: proven safety and efficacy, cost effectiveness, and at least the same effectiveness with lower morbidity compared to existing therapeutic options.

Cryosurgery meets these criteria and has demonstrated feasibility, tolerance, and effectiveness in destroying breast tumors. It offers a simple procedure with low morbidity, reduced or absent pain, and cost effectiveness [11,12]. Cryosurgery not only destroys cancer cells but also induces the release of intact tumor-specific antigens into the circulation, which is vital for immune system recognition and response [13]. Studies have shown that cryosurgery can stimulate a tumor-specific immune response, which is reflected in the regression of metastatic lesions. The presence of higher levels of tumor-infiltrating lymphocytes (TILs) has been associated with better response rates to neoadjuvant chemotherapy and improved survival rates. Animal studies have demonstrated that cryosurgery increases immune effector cell numbers while decreasing immunosuppressive regulatory T cell (Tregs) numbers associated with tumor growth promotion.

Cryosurgery is typically performed in an outpatient setting under local anesthesia, resulting in reduced sedation requirements, operating room needs, surgical complications, recovery times, and healthcare costs. Importantly, it offers an attractive alternative to surgery with better cosmetic outcomes. Unlike surgery, cryosurgery does not require excision within the breast parenchyma, which can cause breast asymmetry and compromised cosmetic results. When analyzing potential cryosurgery-associated complications, major complications are classified as Common Terminology Criteria for Adverse Events (CTCAE) grade 3 or more and minor complications as CTCAE grade 1–2. Major complications include major arterial bleeding after cryoprobe removal and skin necrosis or pectoralis muscle necrosis, and minor complications include minor bleeding, pain, minor skin freeze burns, localized edema, swelling, ecchymosis, skin induration, pruritis, mild hematoma, seroma, and nodular thickening at the cryoablation site.

Meta-analyses comparing various ablation modalities, including cryoablation, radiofrequency ablation (RFA), microwave ablation (MWA), high-intensity focused ultrasound (HIFU), and laser ablation, have shown that cryoablation achieves satisfactory to excellent cosmesis in over 95% of treated patients, with lower complication rates compared with other modalities (5% vs. 18%) [14]. Minor adverse events associated with cryoablation, such as minor bleeding, pain during anesthetic injection, and bruising, are similar to those associated with breast needle biopsy. Large randomized controlled trials are still needed to evaluate cryoablation’s long-term benefits, considering factors such as breast density category or the biological subtype of the tumor in terms of treatment response.

Comparisons of different minimally invasive thermal therapies have shown that cryoablation can achieve complete tumor ablation in 36–83% of breast cancer patients treated with cryoablation, 76–100% in RFA, 20–100% in HIFU, 13–76% in laser ablation, and 0–8% in MWA, highlighting cryoablation as a new promising tool for the local destruction of small breast primary or residual persisting tumors after systemic therapy [15].

Alternatively, cryoablation can be utilized as a salvage method for treating local recurrence following conservation therapy. However, to ascertain the comparable efficacy of cryoablation to breast conservation treatment, it is crucial to conduct large-scale, multi-center randomized control studies that evaluate its long-term benefits. Interestingly, a study comparing cryosurgery with other modalities, such as radiofrequency ablation (RFA), microwave ablation (MWA), and radiation therapy, discovered that ultra-low temperatures induced a stronger immune response. This heightened response could be attributed to the ability of heat-based techniques to cause protein denaturation and antigen damage [16,17].

Furthermore, Fine et al. [9] conducted a study using the N_2_-based ProSense Cryosurgical System (IceCure Medical Ltd., Caesarea, Israel) in women aged 60 years or older. The study reported no severe device-related adverse complications. However, it did identify that two-thirds of the overall moderate complications, found in 2.4% of the patients, and mild complications, found in 18.4% of the patients, were unrelated to the study procedure or device. Instead, these complications were specific to the patients and associated with factors such as age and comorbidities, including stroke, respiratory failure, urinary tract infection, abdominal pain, headache, and pneumonia.

**Figure 4:**
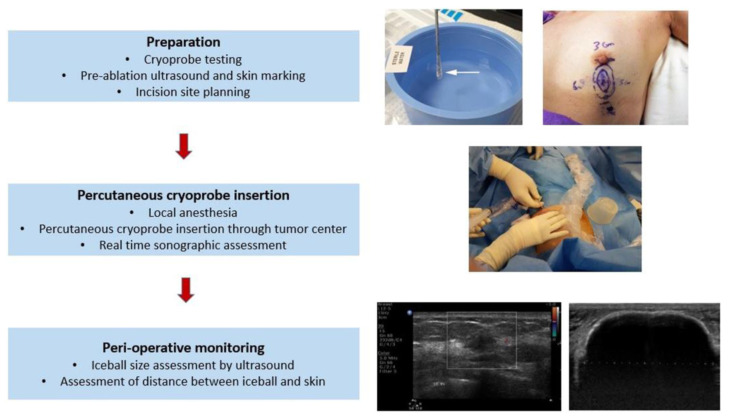
Schematic representation of the cryosurgery procedure in breast cancer from preparation to peri-operative monitoring [18].

Figure 4 provides a schematic representation of the cryosurgery procedure for breast cancer, illustrating the various stages from preparation to peri-operative monitoring.

**Figure 5:**
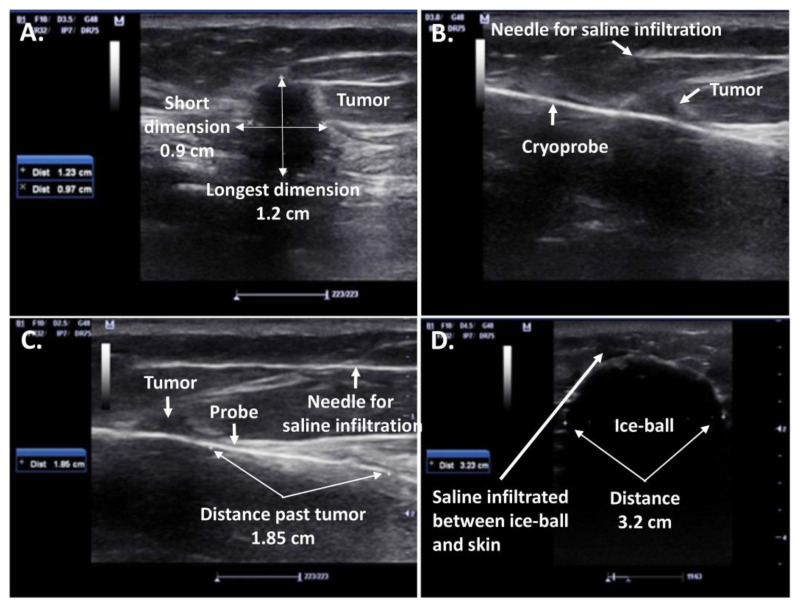
Ultrasound (US)-guided cryosurgery for a 1.2 cm diameter right breast IDC: (**A**) Size of tumor visualized by ultrasound; (**B**) Visualization of cryoprobe penetration hydro-dissection with saline; (**C**) Visualization of distance of probe projection past the tumor; (**D**) Transverse dimension of the ice-ball (3.2 cm) completely engulfing the transverse dimension of the tumor (0.9 cm), generating at least a 1 cm safety freeze margin on either side of the tumor [22].

The cryosurgery procedure is performed with a single cryoprobe or multiprobes placed percutaneously into the lesion under the guidance of an ultrasound scan (US) or magnetic resonance imaging (MRI). As a result, an ice ball around the cryoprobe is formed, covering the entire tumor with an appropriate surrounding tissue margin. According to experimental studies, efficient cryoablation could be achieved with an 8–10 mm margin as proposed by Roubidoux et al. [19], while histology findings of a study on an animal model by Rabin et al. [20] demonstrated that a narrower 5 mm margin is sufficient in the cryoablation of a breast tumor. The ablation procedure consists of a first freeze, a passive thaw, and a second freeze, with times depending on the size of the targeted lesion, the ablation margin, and the device [21]. Saline hydro-dissection, demonstrated in Figure 5, is an additional procedural aspect required for overlying skin protection from the ultra-low temperatures of the ice ball.

Surgical preparation is vital, and eligibility criteria for cryoablation must be considered. Ideal candidates are well-visualized low-grade invasive ductal carcinomas (IDCs) < 1.5 cm without an extensive intraductal component (EIC), located 1 cm from the skin, that are lower grade, less aggressive tumors, less likely to be multicentric, multifocal, and contralateral. Patients with a pure IDC or ductal carcinoma in situ (DCIS) with an EIC are usually excluded due to the lack of US correlation and their potential extension outside of the ablation zone, respectively. Similarly, an invasive lobular carcinoma (ILC) is typically excluded from cryoablation as its extent could be underestimated by imaging. Cryoablative success followed by surgical resection in 100% of IDCs of size ≤ 1.0 and ≤1.5 cm was reported by Sabel et al. [23], whereas the modality was not found to be successful for tumors > 1.5 cm. Similarly, a phase II study of invasive breast cancers by the American College of Surgeons Oncology Group (ACOSOG) showed 93.8% success rate for tumors < 1 cm and 88.7% for low-grade tumors, while showing 75.9% success for patients with a tumor < 2 cm [24]. Nevertheless, it should be highlighted that the cryoablation success in tumors > 1.5 cm was, to a great extent, affected by a failure of imaging to exclude patients with adjacent occulted disease. In addition, taking into account cases of incomplete and misplaced ablation, more trials with technique modifications and patient selection are required to improve modality success.

**Figure 6:**
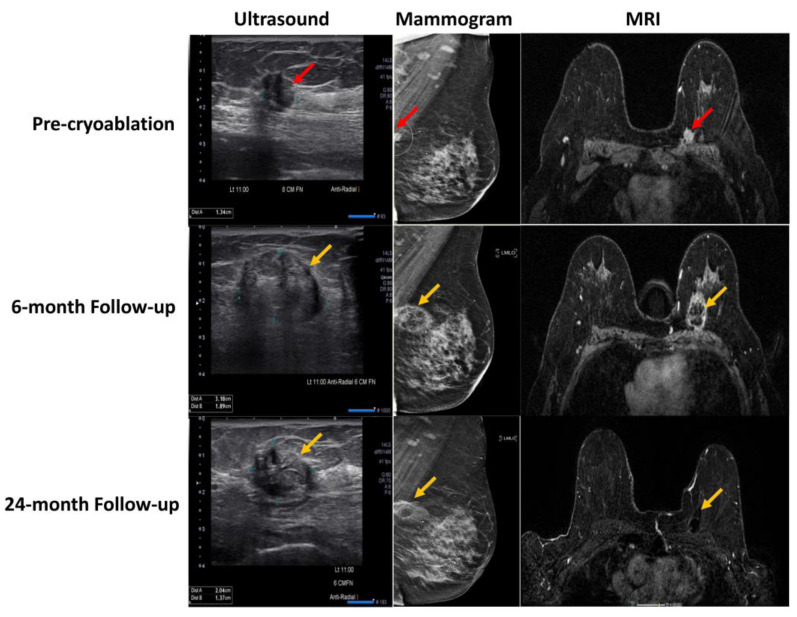
Pre-cryoablation and post-cryoablation imaging at the 6- and 24-month timepoint by US, mammogram, and MRI of a patient with invasive ductal carcinoma (red arrow) in the left breast and an identified area of fat necrosis (yellow arrow) [22].

Pre-treatment surgical design is also vital in order to achieve complete tumor ablation, considering tumor orientation, cryoprobe insertion along the long axis, and understanding the size and shape of the ice ball that would be formed and the number of freezing cycles needed [18]. Since the size of tumor is an essential predictor of residual cancer, pre-cryosurgery US, MRI, and mammography assessment in addition to multiple core biopsies of the tissues that surround the tumor are always required for optimal patient selection and the exclusion of extensive intraductal components, which could potentially lead to an incomplete tumor eradication by cryosurgery. At the same time, the imaging modalities, especially breast MRI, play a crucial role in post-cryoablation follow-up in determining the zone of fat necrosis, as depicted in Figure 6.

A supportive multi-institutional prospective study with no recorded complications conducted by Sabel et al. [23] showed 93% efficiency, with 85% of patients showing no residual viable tumor. It demonstrated that the degree of residual tumor after cryoablation depends on the tumor size and the presence of an intraductal component, as this modality was not effective in tumors > 1.5 cm and with in situ components. The aforementioned results were supported by the Adachi et al. [25] study on ductal carcinoma and DCIS with sizes  ≤ 1.5 mm, which demonstrated only 0.5% recurrence, overall highlighting a successful rate of cryosurgery and low rates of local recurrence at the 1-year follow-up evaluation in patients with low-risk, early-stage breast cancer. The retrospective study limitations included non-uniform intervals between cryoablation and imaging, which resulted in potentially different findings between the follow-up phases. This should be addressed in larger prospective studies with strict timing of follow-ups and enough recurrences to investigate the modality efficiency in restaging after breast cryoablation. A study by Littrup et al. [26] investigating both early- and late-stage breast cancers with mean lesion diameters of 1.7 ± 1.2 cm showed no significant complications, and, most importantly, there were no local cancer recurrences during follow-up periods lasting 18 months. In addition, the therapy was well tolerated and patients exhibited no breast distortions. Nevertheless, the major limitation of this study arises from its limited nature, with a self-selected patient group that required vigilant skin protection, dictating variation of the technique according to tumor location and size. Pfleiderer et al. [27,28] suggested that for tumors > 1.5 mm, multiple cryoprobes are required to achieve larger ice balls, while cryoablation is not an efficient treatment modality for use in pre-invasive disease of the breast as residual cancer was recorded in patients with DCIS. Similarly, Machida et al. [29], with a mean follow-up of 40.6 months on 54 patients with ductal carcinoma in situ or invasive carcinoma ≤ 1.5 cm without subsequent surgical resection, showed that only one patient experienced ipsilateral breast cancer recurrence. A study by Roubidoux et al. [19] showed that two of nine patients had residual disease, which, in one case, had one small invasive focus, and in another, an extensive multifocal ductal in situ neoplasm, overall highlighting the importance of the precise patient selection aspect in cryoablation. In a prospective single-arm clinical trial on early breast cancers, Kwong et al. [18] found a less than satisfactory high residual cancer rate of 46.7% in tumors > 1.5 cm, demonstrating that it is prudent to conduct cryoablation in tumors ≤ 1.5 cm and in non-luminal cancers and, therefore, that careful pre-operative assessment and intra-operative monitoring is vital to ensure complete breast tumor cryoablation.

Interestingly, cryosurgery has been found to be a practical alternative modality to pre-operative wire localization, facilitating more accurate and easier resection and decreasing positive margin rates during lumpectomy of small and non-palpable breast cancers [30]. Similarly, Tafra et al. [31] demonstrated the efficiency of cryo-assisted localization (CAL) as an alternative to hook-wire localization, resulting in cancer-cell-free margins and a lower volume of the resected breast parenchyma compared with the traditional technique. In a further study, Tafra et al. [6] showed a significantly lower volume of the specimens with a similar positive margin status in the CAL group compared with the needle-wire localization group, in addition to the recorded ease of use, better quality of the specimen, patient satisfaction, and short term cosmesis, overall demonstrating CAL as a promising device to aid lumpectomy and to tailor the amount of tissue resected, leading to a more precise lumpectomy. Nevertheless, the CAL group showed lower invasive cancer positive margin rates with higher observed ductal-carcinoma-in situ-positive margin rates; therefore, further larger studies to evaluate the technology are needed.

The abovementioned significant progress in breast cancer genomics provided a better understanding of tumor cryoablation at a molecular level and, consequently, of the prognosis, while allowing more precise patient-specific approaches, and it therefore led to a reconsideration of how to manage elderly patients in the light of a quicker pace of population ageing than ever. Cryosurgery as a minimally invasive ablation technique has been found to achieve efficacy equal to that of breast conservation therapy, while avoiding the risk associated with surgical intervention. A study of a 3-year interim analysis by Fine et al. [9] evaluating women aged 60 years and older with unifocal, invasive ductal carcinoma size ≤ 1.5 cm classified as low to intermediate grade showed cryoablation as efficient and safe with similar to surgical standards of care and local control, while avoiding the associated surgical risks. Nevertheless, a follow-up assessment up to and beyond the primary 5-year end point is required to confirm the findings with standardized adjuvant therapies to minimize the potential confounding effects of variations on the study results. Another study on the subject by Habrawi et al. [22] on patients over 60 years old with diagnoses of infiltrating ductal carcinomas size ≤ 1.5 cm demonstrated good tolerance, no serious complications, no cosmetic deficits, and no evidence of disease recurrence, suggesting that early breast cancers up to 1.5 cm in elderly patients with a favorable low-risk profile could benefit from a single session of cryoablation without the need for subsequent surgical intervention. Similar to other studies, this series with a small number of patients and a short follow-up requires further long-term follow-up with a larger sample size to record local control. A study on larger ductal invasive unifocal breast cancers ≤ 2 cm in post-menopausal women between 64 and 82 years reported the successful destruction of the target lesion in 14 of the 15 cases, highlighting the efficiency of cryoablation of single small breast cancers, resulting in complete necrosis, good cosmetic outcome, and patient satisfaction [32].

### 2.2. Cryosurgery Potential in Metastatic Breast Cancer

To date, the majority of cryosurgery research has focused on patients with early-stage small breast cancers, while its application in patients with metastatic breast cancer remains limited. The therapeutic options for metastatic breast cancer, also known as stage IV breast cancer that has spread to other organs such as the bones, liver, brain, and lungs, depend on various factors, including the patient’s estrogen receptor (ER) or human epidermal growth factor receptor 2 (Her2) status, gene mutations, metastatic locations, previous treatments, and involve systemic drugs and local and regional treatments, including surgery, chemotherapy, targeted biological therapy, immunotherapy, and/or radiation therapy. Recent evidence suggests that locoregional treatment of primary breast cancer after primary systemic therapy in patients with stage IV disease improves overall survival [33]. While lumpectomy has shown survival benefits for selected patients, resection of the primary tumor could remove potential tumor stem-cell sources that support distant metastases by different neoplastic cell lines. However, surgery may also expose the patient to surgical complications and delay systemic treatment [34].

Locoregional therapy of the primary tumor in de novo stage IV breast cancer has been reported to improve overall survival (OS) and reduce mortality risk [33,35,36,37]. Nevertheless, due to the limited research and conflicting studies, cryosurgery, as a minimally invasive modality with potential antitumoral immune effects, has gained relevance for patients with stage IV metastatic disease. Cryosurgery may play a role in controlling the development of metastases. A retrospective study by Niu et al. [10] analyzed the therapeutic effects of cryosurgery in combination with other therapies such as immunotherapy and chemotherapy in metastatic patients after failed radical surgery and reported the highest median OS of 83 months in the cryo-immunotherapy group, highlighting the overall beneficial effect of cryosurgery when used in conjunction with other treatment modalities. Furthermore, a retrospective study by Pusceddu et al. [38] demonstrated that cryoablation of primary advanced breast cancers in patients with bone metastatic ductal invasive breast lesions, who had previously received systemic therapy, is an effective, feasible, and well-tolerated treatment option. Complete regression was achieved in 88% of patients, but further large-scale studies are warranted. Supporting this perspective, Pusceddu et al. [39] also demonstrated the safety and efficacy of cryoablation when ablating primary tumors in stage IV breast cancer patients, achieving complete tumor necrosis in 85.7% of patients at the 2-month follow-up and 100% at the 6-month follow-up. However, given that the study, like almost all breast ablation studies, was retrospective in nature, prospective well-designed studies comparing cryoablation with other treatment options in metastatic breast disease are essential for a deeper understanding of the role of cryosurgery in this subgroup of patients and its potential survival benefits.

### 2.3. Cryosurgery and Immunotherapy

In addition to the release of intact tumor-specific antigens into the circulation for subsequent recognition and response by the immune system, there is a crucial mechanism that operates to balance immune system activation, preventing dysregulation of the response and the associated autoimmunity. This process is mediated by cytotoxic T lymphocyte-associated protein 4 (CTLA-4), also known as the immune checkpoint receptor, which is found on T cells and binds to B7 on antigen-presenting cells (APCs), leading to the downregulation of the clonal expansion of T cells. Another critical checkpoint is programmed cell death 1 protein (PD-1) in T cells, which binds to programmed cell death ligand 1 (PD-L1) in peripheral tissues, resulting in inhibition of the immune response and the promotion of self-tolerance. The understanding of immune checkpoints has led to the development of immune checkpoint blockade agents for cancer treatment, which remove the brakes on the immune system, allowing for continued antigen-specific recognition and response.

However, immunotherapy agents have shown limited efficacy in breast cancer management due to a low mutational tumor burden and the lack of T cell-specific peptides presented to the T cells by the tumor. Therefore, the combination of cryoablation and immunotherapy, by leveraging checkpoint inhibitors and the ability of ultra-low temperatures to induce tumor-specific immune responses, is believed to enhance the efficacy of immunotherapy in breast cancer. This synergistic approach has been found to be associated with the activation of de novo adaptive immune response components critical for tumor antigen release and presentation, the reduction of immune suppression, and the activation of tumor antigen-specific T cells. Achieving an optimal immune response to tumors is therefore accomplished through cryoablation-induced tumor antigen release combined with immunologic agents like ipilimumab, leading to reduced immune suppression and increased activation of tumor antigen-specific T cells.

A pilot clinical study investigating the synergism of cryoablation and pre-operative single-dose ipilimumab (anti-CTLA-4) in early-stage breast cancer patients demonstrated the safety and, importantly, the favorable systemic immunologic and intra-tumoral effects of this combination. These effects included higher inducible costimulator (ICOS) expression, which plays a crucial role in increased antitumor activity [40]. The study also showed continuous proliferation of CD4 and CD8 cells. Similar conclusions were highlighted in a study by Page et al. [41], which focused on T cell clonality and intra-tumoral T cell density. The study demonstrated that cryoablation induced the death of both tumor cells and tumor-infiltrating lymphocytes (TILs), leading to the release of a broader variety of tumor-specific antigens required for immune system recognition. The synergistic effect of cryosurgery and immunotherapy mediated the proliferation of a small subset of T cell clones. Despite promising findings, research on the synergism of cryoablation and immunotherapy is still limited by the use of animal models with small sample sizes. Several ongoing clinical trials aim to examine this relationship, potentially targeting not only small breast cancers but also distant metastatic disease.

One of these trials focuses on the number of adverse events as the primary outcome of cryoablation, Nivolumab (anti-PD-L1), and Ipilimumab (anti-CTLA-4) treatment in patients with early-stage breast cancer [42]. Another phase II trial is investigating the impact of pre-operative cryoablation, Nivolumab, and Ipilimumab treatment in patients with triple-negative breast cancer after neoadjuvant chemotherapy [43].

A phase I clinical trial is currently underway to investigate the feasibility and side effects of combining cryoablation, Nab-paclitaxel, and Atezolizumab (an anti-PD-L1 drug) for the treatment of patients with metastatic triple-negative breast cancer [44]. Promising results from these ongoing trials would significantly enhance the discourse surrounding the potential of synergistic treatment approaches involving cryoablation and immunotherapy for breast cancer.

### 2.4. Cryosurgery and DCIS

The management of ductal carcinoma in-situ (DCIS) is a subject of controversy due to the possibility that many diagnosed cases may never pose a life-threatening risk. This concern raises the issue of potential overtreatment through surgery for certain patients. As an alternative approach, active surveillance has been suggested as a management strategy, especially for low-grade DCIS [45]. However, accurately predicting which cases of DCIS will never progress to invasive disease is difficult, which could result in the undertreatment of a significant number of women. Consequently, it is worth exploring cryoablation as a potential alternative to surgical resection or active surveillance for DCIS, given that it is feasible with proper patient selection. Further investigation is required to assess the efficacy of this approach in patients diagnosed with clearly delineated DCIS through imaging techniques. The introduction of mammary ductoscopy has recently provided the means for the direct visualization of DCIS lesions using an endoscope inserted through the nipple. The advancement of a 1 mm cryotherapy probe would enable the ablation of DCIS lesions identified through mammary ductoscopy [46]. Although several studies are currently underway, additional research on pure DCIS cases of any size is required due to the lack of data on the efficacy of cryosurgery in these patients.

## 3. Conclusions

Cryotherapy shows promise as a technique for ablating primary tumors in patients with T1 breast cancer and limited intraductal components, as determined by breast imaging, including breast MRI. Cryoablation has been found to induce a systemic tumor-specific response, thereby enhancing the tumor’s susceptibility to immunotherapy agents such as checkpoint inhibitors and anti-Her2 antibodies in triple-negative and Her2-positive breast cancer, respectively. Since neoadjuvant systemic therapy is the optimal treatment for these molecular subtypes, cryotherapy should be further investigated in future trials as an alternative to surgery for ablating the primary tumor site while the patient is receiving immunotherapy.

For patients presenting with de novo stage IV breast cancer, cryotherapy could be considered as the preferred local ablation treatment for the primary tumor, as it avoids invasive surgical intervention and may induce a systemic immune response against the tumor elsewhere in the body. Cryotherapy also holds potential for eradicating oligometastatic disease in organs such as the liver, providing an alternative to complex invasive liver resection surgery.
